# Mendelian randomization indicates causal effects of estradiol levels on kidney function in males

**DOI:** 10.3389/fendo.2023.1232266

**Published:** 2023-12-19

**Authors:** M. Kamal Nasr, Claudia Schurmann, Erwin P. Böttinger, Alexander Teumer

**Affiliations:** ^1^ Institute for Community Medicine, University Medicine Greifswald, Greifswald, Germany; ^2^ Digital Health Center, Hasso Plattner Institute, University of Potsdam, Potsdam, Germany; ^3^ DZHK (German Centre for Cardiovascular Research), Partner Site Greifswald, Greifswald, Germany; ^4^ Department of Psychiatry and Psychotherapy, University Medicine Greifswald, Greifswald, Germany; ^5^ Hasso Plattner Institute for Digital Health at Mount Sinai, Icahn School of Medicine at Mount Sinai, New York, NY, United States

**Keywords:** glomerular filtration rate, steroids, albuminuria, genome-wide association study, causality

## Abstract

**Context:**

Chronic kidney disease (CKD) is a public health burden worldwide. Epidemiological studies observed an association between sex hormones, including estradiol, and kidney function.

**Objective:**

We conducted a Mendelian randomization (MR) study to assess a possible causal effect of estradiol levels on kidney function in males and females.

**Design:**

We performed a bidirectional two-sample MR using published genetic associations of serum levels of estradiol in men (*n* = 206,927) and women (*n* = 229,966), and of kidney traits represented by estimated glomerular filtration rate (eGFR, *n* = 567,460), urine albumin-to-creatinine ratio (UACR, *n* = 547,361), and CKD (*n* = 41,395 cases and *n* = 439,303 controls) using data obtained from the CKDGen Consortium. Additionally, we conducted a genome-wide association study using UK Biobank cohort study data (*n* = 11,798 men and n = 6,835 women) to identify novel genetic associations with levels of estradiol, and then used these variants as instruments in a one-sample MR.

**Results:**

The two-sample MR indicated that genetically predicted estradiol levels are significantly associated with eGFR in men (beta = 0.077; *p* = 5.2E-05). We identified a single locus at chromosome 14 associated with estradiol levels in men being significant in the one-sample MR on eGFR (beta = 0.199; *p* = 0.017). We revealed significant results with eGFR in postmenopausal women and with UACR in premenopausal women, which did not reach statistical significance in the sensitivity MR analyses. No causal effect of eGFR or UACR on estradiol levels was found.

**Conclusions:**

We conclude that serum estradiol levels may have a causal effect on kidney function. Our MR results provide starting points for studies to develop therapeutic strategies to reduce kidney disease.

## Introduction

Chronic kidney disease (CKD) due to impaired kidney function is a major contributor to death and suffering in the 21st century (1), affecting an estimated 843 million individuals worldwide in 2017. Between 1990 and 2017, the global all-age mortality rate attributed to CKD increased by 41.5%. Studies and research continue to be conducted to identify and evaluate the risk factors associated with the development of CKD. These risk factors include high blood pressure and diabetes mellitus type 1 and 2 (1).

Furthermore, sex-associated differences in the epidemiology of kidney disorders have been observed (2, 3). Studies and trials have shown that most people who reach end-stage kidney disease (ESKD) are men, and with a faster disease progression than women (4, 5). However, randomized controlled trials assessing a causal effect of estradiol levels on kidney disease are lacking.

Several theories exist to explain the sex-associated differences in exposure and prognosis of kidney disease. These include unhealthy lifestyle and habits, which are found to be more prevalent in men than in women (2, 6). However, one important physiological difference is the steroidal sex hormones, including testosterone and estradiol, which play an essential role in the development of sexual characteristics (7).

Similar to the testosterone levels in men, estradiol levels in women can vary depending on age and menstrual status. In premenopausal women, estradiol levels vary throughout the menstrual cycle starting from 20 pg/mL to 80 pg/mL during the early phase of menstruation (8), followed by a gradual increase until the level reaches its maximum at the middle of the cycle, before decreasing again at the end of it. The estradiol levels could reach 300 pg/mL by the end of the second week of the menstruation cycle (8, 9), with an upper limit even reaching higher than 600 pg/mL. Estradiol levels are significantly lower in postmenopausal women. Some studies report average levels between 50 pg/mL and 120 pg/mL in older women who are no longer menstruating (8, 9).

Sex hormones have secondary functions such as organ development and prevention of disorders, such as osteoporosis (10). However, their impact on kidneys is not fully understood. Studies have shown an association between lower testosterone levels in men and increased all-cause mortality risk at advanced stages of CKD (11). A significant association between dialysis and decreased estradiol levels was also found in women, resulting in the lack of ovulation and abnormal menstruation cycles (12). The complexity of the regulation of the estradiol hormone in women has made it difficult to study its role and association with kidney functions. However, studies using animal models, which were designed to uncover the reasons underlying this association, suggested that estradiol and other estrogens could have a nephroprotective effect by antagonizing apoptosis of the podocytes, especially in females (13, 14). Estradiol has also shown protective effects on other kidney-damaging pathways like nitrogen oxide production and collagen synthesis. On the other hand, testosterone has shown destructive effects on kidney function, by either inducing apoptosis of podocytes or other mechanisms such as fibrosis of kidney cells (2, 15). Most of these studies were conducted in animal models, thus creating a need to investigate the effect of sex hormones on kidney functions in humans (16).

Randomized controlled trials are a well-established method to assess causality. However, the high cost of conducting these studies and their challenging feasibility are pertinent drawbacks of this approach (17). Mendelian randomization (MR) is an alternative method using genetic associations as instrumental variables to overcome possible bias due to confounding when drawing causal inference from observational studies (18). MR methods have been utilized to investigate causal effects of several phenotypes, including kidney function (19–22).

Previous studies conducting MR analyses of sex hormones on kidney function focused on testosterone and sex hormone-binding globulin (SHBG) by using data from the UK Biobank cohort study (21, 22). These data revealed that genetically predicted SHBG levels are associated with a protective effect on kidney function and a reduced risk of CKD in the male population (21). In addition, genetically predicted testosterone levels increased the risk of CKD in men (22).

However, there is a lack of studies investigating the causal effect of estradiol hormone levels on kidney function in both male and female populations (23). Estradiol levels are subject to wide intra-individual variation in the premenopausal female population, whereas they are generally lower in postmenopausal women, and often below the detection limit in men. These variations complicate the identification of genetic variants that are significantly associated with the hormone levels (23, 24).

Here, we conducted a bidirectional MR to assess causality between the levels of the estradiol sex hormone and kidney traits in both males and females, using known and novel estradiol-associated genetic variants as instruments. The significant findings of the two-sample MR were aimed for validation by additional pleiotropy-robust MR methods and a one-sample MR using the UK Biobank cohort study data.

## Materials and methods

### Study design

We applied MR to assess causal associations of the estradiol hormone on the urine albumin-to-creatinine ratio (UACR), the estimated glomerular filtration rate (eGFR) based on serum creatinine, and CKD using two-sample MR analyses. We included the single-nucleotide polymorphism (SNP) summary statistics for males (*n* = 206,729) and females (*n* = 229,966), from genome-wide association studies (GWAS) of two different publications conducted in the UK Biobank, for instrument selection of estradiol in the two-sample MR. We used the summary statistics on kidney-related traits obtained from the CKDGen Consortium (25, 26). The datasets were limited to individuals of European ancestry aligning them with the estradiol sample population. The GWAS included 480,698 individuals for CKD (41,395 cases), 567,460 individuals for eGFR, and 547,361 individuals for UACR. In these studies, both eGFR and UACR were log-transformed. Additionally, the UACR was inverse-normal transformed before conducting the GWAS.

**Figure 1:**
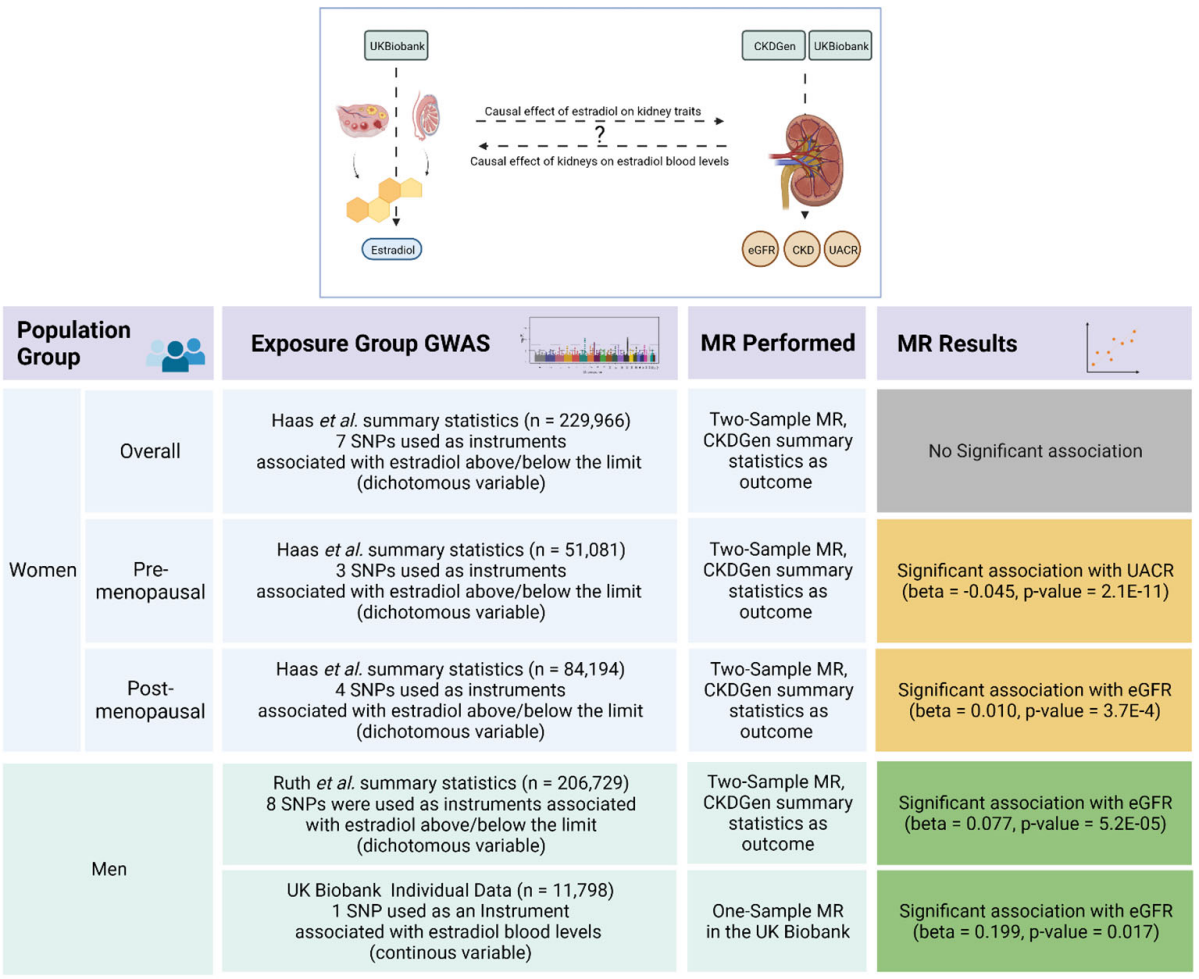
Overview of the Mendelian randomization analyses and its results.The upper scheme illustrates the main goal of our Mendelian randomization (MR) study investigating a possible causal effect between estradiol levels and kidney function traits as represented by estimated glomerular filtration rate (eGFR), urinary albumin-to-creatinine ratio (UACR), and risk of chronic kidney disease (CKD). The lower table summarizes the strata, datasets, methods, and the results of the MR analyses. The green boxes represent validated significant results, where the results in the yellow boxes could not be confirmed by sensitivity analyses. The figure was created with BioRender.com.

To validate and test the robustness of the significant two-sample MR findings, we conducted a GWAS on the continuous estradiol levels in the UK Biobank dataset as a means to discover instruments for a subsequent one-sample MR. Finally, we tested for a potential causal effect of the kidney traits on estradiol levels. An overview of the analyses performed, including its main results, is provided in **Figure 1**.

To ensure that the instruments were independent from each other, we used LocusZoom (https://my.locuszoom.org) with an *R* ^2^ < 0.01 cut-off value to select the variant with the smallest *p*-value per locus (27).

### Dataset selection for the two-sample Mendelian randomization

We applied a two-sample MR, which can use SNP–outcome and SNP–exposure associations obtained from the GWAS datasets, to assess causality. Three core assumptions on genetic variants have to be fulfilled to act as suitable instruments in a MR analyses (1): association with the exposure (2); independence of the outcome given the exposure and all the confounders of the exposure–outcome association; and (3) independence of the factors that confound the exposure–outcome relationship (28). We used the datasets and applied the methods described in detail below to ensure the validity of the instruments as far as possible. For the exposure data, we used two different published GWASs that have investigated genetic associations with estradiol. We chose instruments with genome-wide significant associations (*p* < 5*10^–8^) with the exposure, and removed variants with pleiotropic effects on the potential confounders, as described below. Finally, we applied Mendelian randomization pleiotropy residual sum and outlier (MR-PRESSO) analysis (29) to identify outliers among the instruments, which were then removed prior to the subsequent MR analyses.

The results from Haas et al. (30) included women of European ancestry stratified by their menstruation status. The second source for the genetic instruments was obtained from the study by Ruth et al. (23), in which a GWAS in men with European ancestry was conducted. Both studies used data from the UK Biobank cohort study and identified estradiol as a dichotomous variable being above the detection limit (23, 30). The selection of potential instrumental variables was performed by following the guidelines for MR analyses (31).

To assess the causal effects of estradiol levels on kidney function, we used genetic predictors of log-transformed UACR and eGFR from the CKDGen Consortium GWAS results as exposure (25, 26). As the log-transformed UACR was included as sex- and age-adjusted inverse-rank-normalized residuals in the GWAS, the unit change corresponds to one standard deviation (SD) change of the log-transformed UACR. We used the generated summary statistics of the GWAS in the UK Biobank for the genetic associations with the log-transformed estradiol levels as an outcome in males and females.

We looked for proxy SNPs with an *R*² value > 0.8 if the potential instrument was not available in the outcome GWAS results. However, no proxies could be found. All instruments were primarily associated with the exposure according to the Steiger test (32). The results were subsequently verified for no association with body mass index (BMI), body fat mass, and type 2 diabetes as potential confounders using the PhenoScanner webtool (33). Details on the instrument selection are provided below.

### Details on variant selection in the published datasets used as instruments for estradiol

The first data source was obtained from Haas et al. (30). Of the 229,966 women with European ancestry available in the UK Biobank dataset, 51,081 were premenopausal and 84,194 were identified as being postmenopausal. The GWAS was conducted on estradiol as a continuous phenotype (inverse-rank normalized), and as a dichotomized outcome using PLINK 2.0 (34). The GWAS on the continuous trait revealed only one significant association (rs727428), which also represented a known association with SHBG and testosterone levels (35), and was thus not treated as a valid instrument for estradiol. Therefore, we only used the results of the dichotomized trait for the subsequent MR analyses. We extracted all the independent SNPs with genome-wide significance (*p* < 5*10–^8^) presented in Haas et al. The GWAS results for women overall provided 10 SNPs with a significant genetic association with estradiol, only seven of them were suitable candidates for the following two-sample MR. Two SNPs (rs774021038 and rs71181755) were excluded, as there were no available corresponding results in the outcome summary statistics, while one SNPs (rs34929649) was excluded due to its association with the fat mass of body parts. Of the selected seven SNPs, four SNPs were eligible as candidates in the postmenopausal women group and three in the premenopausal women.

The second source for the genetic instruments for the two-sample MR was obtained from the study by Ruth et al. (23), in which a GWAS in the UK Biobank cohort was conducted. In that GWAS, genetic variants associated with estradiol levels in men above vs. below the assay detection limit were analyzed using a linear model. The authors identified 22 variants with a statistically significant association with estradiol levels, of which 10 (rs188982745, rs570754094, rs781858752, rs34019140, rs201687269, rs5933688, rs12850857, rs776715248, 4:69958680_GA_G, and 5:35983283_CA_C) were not available in the kidney trait GWAS results, and one SNP (rs117826558) was available only in the summary statistics of the GWAS on CKD.

One SNP, rs1260326 on chromosome 2, was excluded as an instrument because it was significantly associated with eGFR in the CKDGen Consortium summary statistics (*p* = 2*10^–36^) used as outcome, two SNPs were excluded from the analysis due to their association with possible confounders violating the MR assumptions (31): rs45446698 was associated with BMI and fat percentage, and rs727428 due to its association with levels of testosterone and SHBG. MR-PRESSO identified rs657152 as an outlier. This variant is located near *ABO* on chromosome 9, a gene that shows a considerable association with angiotensin-converting enzymes (i.e., ACE1/ACE2) (36) and is supposed to have a direct effect on kidney functionality (37).

All the instruments had a minor allele frequency (MAF) > 1% and a high imputation quality (info score ≥ 0.8) in both the exposure and outcome data. The final list of instruments for estradiol included in the two-sample MR analyses are provided in **Supplementary Tables 1**, **2**.

### Details on variant selection for kidney trait instruments

Of the 256 genome-wide significant associations associated with serum creatinine-based eGFR in the European ancestry sample in the publication of Wuttke et al. (25), 122 variants that were marked with likely support for kidney function and replicated in the MVP study (if available) were included as potential instruments. In total, 14 SNPs were excluded due to their association with BMI or body fat mass (rs10430743, rs10774625, rs10838702, rs112545201, rs11564722, rs1268176, rs2411192, rs35004449, rs3134605, rs3905668, rs55759218, rs632887, rs9375694, and rs9828976), leaving 108 SNPs that passed the selection criteria for instruments (**Supplementary Table 3**).

For UACR, the 63 conditionally independent genome-wide associations of the European ancestry meta-analysis, conducted in the CKDGen Consortium (26), were selected as potential instruments. Five of the SNPs (rs17453832, rs557338857, rs141493439, rs45551835, and rs562661763) were not available in the GWAS of the outcome, leaving 58 instruments for the two-sample MR of UACR (**Supplementary Table 4**).

### Data selection for the GWAS and one-sample Mendelian randomization

The UK Biobank is a prospective cohort study with deep genetic and phenotypic data of more than five hundred thousand individuals recruited from England, Scotland, and Wales (38). Given the large sample size of the UK Biobank study with both estradiol levels and kidney function markers available, we conducted a one-sample MR in this dataset. This additional dataset provided us with the opportunity to assess a causal effect on kidney function, thus extending the published GWASs by using the continuous scale of estradiol levels and restricting the sex hormones to postmenopausal women, which in turn reduced the heterogeneity in these measurements. The details of the sample selection are provided in **Supplementary Figure 1**.

The eGFR was calculated with the CKD-EPI study equation using the R (The R Foundation for Statistical Computing, Vienna, Austria) package “nephro” with serum creatinine levels, age, and sex as inputs, and it was log-transformed for subsequent analyses to match with the two-sample MR.

To identify the SNPs with a significant association as being candidates for instruments, we conducted a GWAS of log-transformed estradiol levels on the imputed genotypes using a linear mixed model implemented in BOLT-LMM (39). The GWAS was conducted for male, female, and sex-combined groups.

For the GWAS and the subsequent one-sample MR, we included only European ancestry individuals (identified by field ID: 22006) with available active consent, genotype data, blood estradiol levels, and creatinine levels measured in urine and blood. We excluded the individuals with a recorded estrogen-based treatment to avoid an exogenous confounding effect (24, 31). For the female population, we included only self-reported postmenopausal women to avoid confounding caused by uncontrolled changes in estradiol levels during the menstruation cycle (8, 9).

Out of 502,505 individuals available in the dataset, 92,810 were excluded because of their non-European ancestry. We excluded 407 individuals due to having estradiol-based treatment (field ID 20003) and 12 individuals due to consent withdrawal. Of the remaining 409,276 individuals, 84,567 premenopausal women and 303,087 individuals with missing estradiol or genotype data were excluded, which resulted in 21,622 individuals (6,835 women and 14,797 men). Of these, 14,797 were male and 11,798 individuals had kidney biomarkers available and were thus included in the subsequent one-sample MR analyses.

We used BMI and age, and also sex in the combined analysis as covariates. In each GWAS, SNPs were filtered using a minor allele frequency (MAF) > 0.001, a Hardy–Weinberg equilibrium *p-value* > 10^–12^, and an imputation info score ≥ 0.8. We used a *p-value* < 5*10^–8^ as a threshold for genome-wide significance. As no instruments in women were found, the one-sample MR was restricted to men.

### Statistical analyses

In the two-sample MR analyses, we used the inverse variance-weighted method (IVW), with multiplicative random effects to assess the causal effect of the exposure on the respective outcome. To test the robustness of the significant MR result, we applied the pleiotropy-robust but less powerful weighted median (40) and MR Egger (41) methods. Cochran’s Q was used to test for the heterogeneity of the causal effect of the individual instruments in the IVW MR. The MR Egger intercept was tested for directional pleiotropy. The analyses were conducted using the R package “TwoSampleMR”.

For the one-sample MR, we applied a two-stage least squares regression implemented in the R packages “tsls” and “ivreg” for UACR and eGFR, and the control function estimator for CKD (28). The analyses were adjusted for age and BMI. **Supplementary Figure 2** provides a schematic overview of the analytical steps performed in the one-sample MR. The power calculation was performed with the “Online sample size and power calculator for Mendelian randomization with a continuous outcome” (https://sb452.shinyapps.io/power/).

For the two-sample MR, a *p*-value < 0.05/4 = 0.0125 was considered statistically significant, correcting for the two different sex strata and the kidney traits included as outcomes, that is, eGFR and CKD for kidney function, and UACR as a marker for kidney damage. For the confirmatory one-sample MR, a *p*-value < 0.05 was considered as significant.

### Ethics statement

In this project only published GWAS summary statistics and the data obtained from the UK Biobank cohort study with ethics approval, as provided on the study website and in the corresponding publication (38), were used.

## Results

### Two-sample Mendelian randomization

The MR using selected known genetic variants as instruments that are associated with estradiol as a dichotomous (above vs. below the assay detection limit) variable in women using the GWAS results of Haas et al. (30) revealed a significant association of genetically predicted higher estradiol levels with a higher eGFR in postmenopausal women (beta = 0.010; *p* = 3.7*10^–4^; **Table 1**). This association was not significant in the premenopausal and overall women groups (**Tables 2**, **3**). The significant association in the eGFR had the effects of similar size and with the same direction in the MR sensitivity analyses, but without reaching the significance level (**Supplementary Table 5**).

**Table 1:** Associations of the inverse variance-weighted two-sample Mendelian randomization of urinary albumin-to-creatinine ratio (UACR), chronic kidney disease (CKD), and estimated glomerular filtration rate (eGFR) in postmenopausal women using the Haas et al. summary statistics for genetically predicted estradiol levels.

Outcome	#SNPs	Estimate/[OR]	95% CI	*p*-value	Q pval
UACR	4	−0.035	−0.076 to 0.007	0.105	0.618
CKD	4	[0.851]	0.651 to 1.052	0.115	0.607
eGFR	**4**	**0.010**	**0.004 to 0.015**	**3.7E-4**	0.814

The Q pval represents the heterogeneity test result p-value. The OR represents the odds ratio of CKD. SNP, number of single nucleotide polymorphism; CI, confidence intervals.

The association results in bold for highlighting statistical significance.

**Table 2:** Associations of the inverse variance-weighted two-sample Mendelian randomization of urinary albumin-to-creatinine ratio (UACR), chronic kidney disease (CKD), and estimated glomerular filtration rate (eGFR) in premenopausal women using the Haas et al. summary statistics for genetically predicted estradiol levels.

Outcome	#SNPs	Estimate/[OR]	95% CI	*p*-value	Q pval
UACR	**3**	−**0.045**	−**0.058 to** −**0.032**	**2.1E-11**	0.942
CKD	3	[0.855]	0.637 to 1.074	0.160	0.471
eGFR	3	0.008	0.002 to 0.014	0.013	0.667

The association results in bold for highlighting statistical significance.

**Table 3:** Associations of the inverse variance-weighted two-sample Mendelian randomization of urinary albumin-to-creatinine ratio (UACR), chronic kidney disease (CKD), and estimated glomerular filtration rate (eGFR) in the overall women population using the Haas et al. summary statistics for genetically predicted estradiol levels.

Outcome	#SNPs	Estimate/[OR]	95% CI	*p*-value	Q pval
UACR	7	−0.032	−0.088 to 0.024	0.262	0.698
CKD	7	[0.915]	0.586 to 1.243	0.595	0.427
eGFR	7	0.007	−0.005 to 0.018	0.270	0.500

The Q pval represents the heterogeneity test result p-value. The OR represents the odds ratio of CKD.

In the premenopausal women group, there were higher genetically predicted estradiol levels significantly associated with a lower UACR (beta = −0.045; *p* = 2.1*10^–11^; **Table 2**). These effect sizes were similar in the sensitivity analyses, but were not statistically significant (**Supplementary Table 6**).

The MR using the instruments for a dichotomous estimation of the estradiol levels in the male population from Ruth et al. revealed that higher genetically predicted estradiol levels are associated with a higher eGFR (beta = 0.077; *p* = 5.2*10–^5^; **Table 4**). Similar results were obtained using the weighted median MR, thus confirming the significant associations (**Supplementary Table 7**).

**Table 4:** Associations of the inverse variance-weighted two-sample Mendelian randomization of urinary albumin-to-creatinine ratio (UACR), chronic kidney disease (CKD), and estimated glomerular filtration rate (eGFR) in males after using the Ruth et al. summary statistics for genetically predicted estradiol levels.

Outcome	#SNPs	Estimate/[OR]	95% CI	*p*-value	Q pval
UACR	7	−0.024	−0.226 to 0.178	0.819	0.243
CKD	8	[0.522]	0.308 to 1.36	0.512	0.492
eGFR	7	**0.077**	**0.040 to 0.114**	**5.2E-05**	0.216

The Q pval represents the heterogeneity test result *p*-value. The OR represents the odds ratio of CKD. The significant results are marked in bold.

No indication of directional pleiotropy or heterogeneity was found for the results (**Tables 1**–**4** and **Supplementary Tables 5**-**7**). The MR scatter plots of the significant associations are given in **Supplementary Figure 3**.

No significant MR results were found for CKD as the outcome (**Tables 1**–**4**).

### Genome-wide association study and one-sample Mendelian randomization

**Figure 2:**
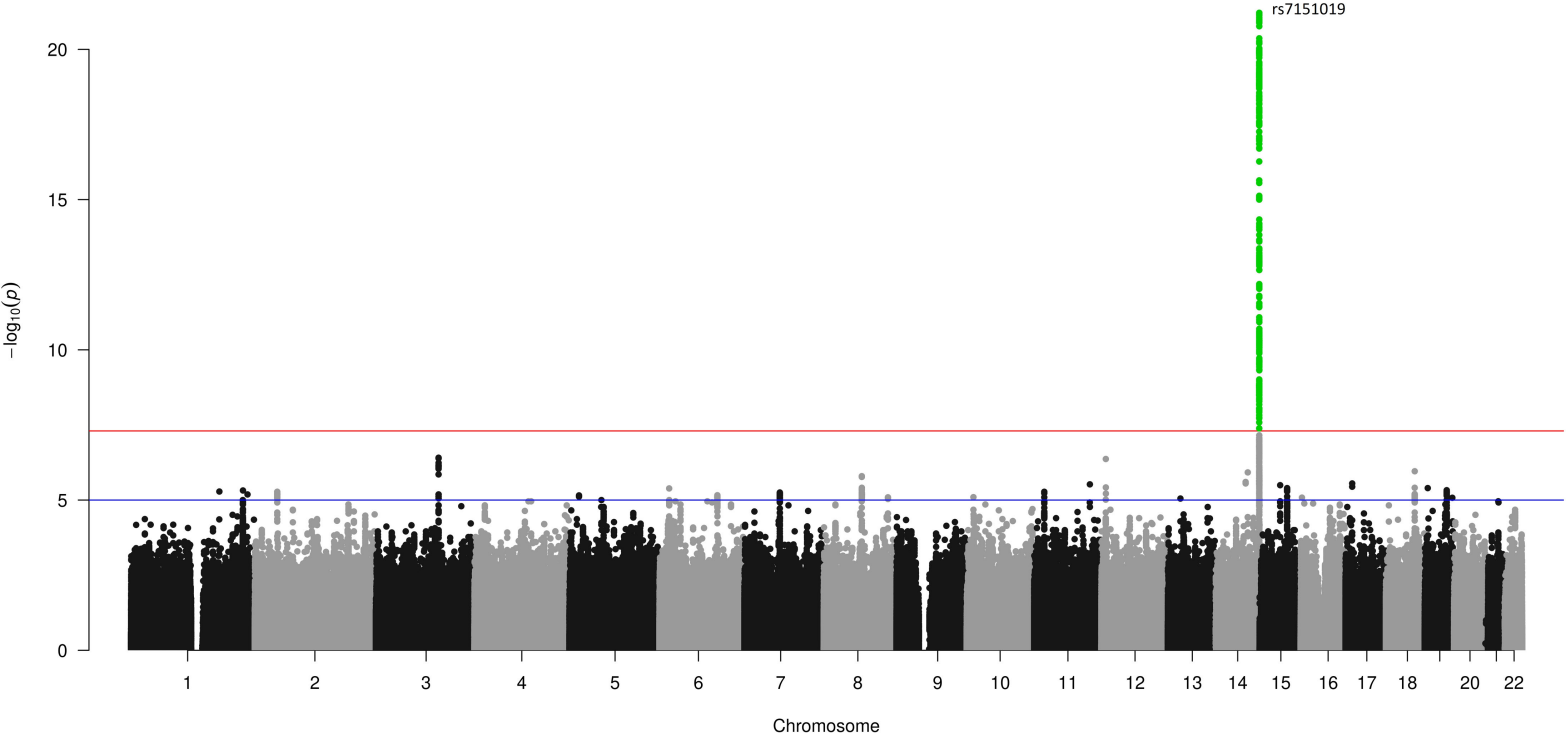
GWAS results for estradiol levels in men in the UK Biobank. A Manhattan plot showing the SNP positions on the *x*-axis, and their association –log_10_ (*p*) on the *y*-axis. The red line represents the threshold for genome-wide significance of 5*10^–8^, the green-colored dots represent variants with a *p*-value equal or smaller than the suggestive threshold of 10^–6^.

The cohort characteristics of the UK Biobank participants included in this analysis are provided in **Supplementary Table 8**. Our three GWASs on continuous estradiol levels in the male (*n* = 14,797), female (*n* = 6,835), and sex-combined (*n* = 21,632) datasets revealed only one genome-wide significant locus (*p* < 5*10–^8^) at chromosome 14 in males (**Figure 2**). The SNP rs7151019 [T/G, MAF = 0.42, beta(T) = -0.026, imputation info = 0.90] represents the variant with the lowest *p*-value at this locus (*p* = 6*10^–22^) explaining 0.73% of the variation of the log-transformed estradiol levels (SD = 0.15). The variant is located close to the immunoglobulin heavy locus (IGH), a protein-coding gene with no known direct link to estradiol metabolism. This locus did not reach statistical significance in the female or in the sex-combined GWAS (**Supplementary Figures 4** and **5**). Of note, this locus was not included in the two-sample MR analyses. The quantile–quantile plots of the GWAS results do not indicate inflation of the *p*-values (**Supplementary Figure 6**). The PhenoScanner (33) did not show an association with BMI, body fat mass, type 2 diabetes, or eGFR. No association in the female or sex-combined samples passed the level of genome-wide significance, thus no MR analyses could be performed in these datasets.

The one-sample MR using the 11,798 males with available kidney biomarkers and the SNP rs7151019 as instrument allowed a detection of at least a 0.3 SD unit change in the kidney trait per SD change in log-estradiol levels at a 80% power. We identified a significant association between estradiol levels and eGFR (beta = 0.199; *p* = 0.017) confirming the two-sample MR results. In concordance with the two-sample MR in males, no significant results were found for CKD and UACR (**Table 5**).

**Table 5:** Associations of the one-sample Mendelian randomization of urinary albumin-to-creatinine ratio (UACR), chronic kidney disease (CKD), and estimated glomerular filtration rate (eGFR) in males of the UK Biobank cohort study.

Outcome	#SNPs	Estimate/[OR]	95% CI	*p*-value
UACR	1	−0.33	−1.170 to 0.389	0.397
CKD	1	[0.758]	0.508 to 1.18	0.206
eGFR	1	**0.199**	**0.036 to 0.362**	**0.017**

The OR represents the odds ratio of CKD. The significant results are marked in bold.

### Effects of kidney function on estradiol blood levels

The MR for testing causal effects of kidney function traits on estradiol levels revealed no significant association of genetically predicted eGFR or UACR with estradiol levels in males or females (**Supplementary Table 9**).

## Discussion

Our two-sample MR analysis based on the instruments assessing estradiol levels below vs. above the detection limit revealed a significant causal effect in males with a positive effect direction, implying that higher levels of estradiol could lead to higher eGFRs. This association was validated in the one-sample MR using continuous levels of estradiol above the detection limit. In addition, such a causal effect on eGFR was suggested by the two-sample MR in postmenopausal women, and an inverse effect in premenopausal women on UACR. However, these results did not reach statistical significance in the sensitivity analyses, and could not be validated using a one-sample MR on continuous outcomes. Overall, the MR analyses suggest a causal effect between higher estradiol levels and better kidney function traits.

To our knowledge, this study is the first MR analysis to investigate the possible causal effect of estradiol levels on kidney function traits. Previous research studies investigated the relationship between the decline in kidney performance in men and women and the change in sex hormone levels in general (13, 42). Based on the results of these studies, researchers sought to identify possible mechanisms of the influence of these hormones on kidney function. For estradiol, most of the studies showed a possible protective effect, either by inhibiting the pathological processes of increasing oxidative stress in the diseased kidney (42), or by inhibiting renal fibrosis aggravation and glomerular sclerosis (13). Animal studies have shown that estradiol plays a protective role by reducing albuminuria and enhancing creatinine clearance (43), which is in line with the effect directions of our MR results. However, these findings were yet not confirmed by sex-stratified analyses. Other studies showed contradicting results. In a nationally representative sample of a United States adult male population, increased levels of estradiol were associated with a decrease in eGFR (44); however, other studies failed to identify an association between endogenous estradiol levels and changes in eGFR or albuminuria (45). Even though most of these studies attempted to find a correlation between sex and the risk of kidney disease, the results of these studies failed to establish a hypothesis for this association.

The aim of our study was to assess a possible causal effect between estradiol levels and kidney function using the MR framework by including summary statistics from published GWASs and data of the large UK Biobank cohort study. To reduce sex-specific heterogeneity in estradiol measurements, we focused on sex-stratified analyses. Our results showed that genetically predicted estradiol levels were significantly associated with an increased eGFR in men. Although we reduced heterogeneity of the estradiol measurements for our two-sample MR analyses in women by using GWAS results that were stratified by pre- and postmenopausal status, our findings were indicative in this sample given the consistent effect direction but not robustly significant. A reason could be the reduced power in the postmenopausal women dataset given the small sample size of individuals above the estradiol detection limit, and the trait variation due to the menstrual cycle in the premenopausal women obtained from the GWAS of Haas et al. (30), where they relied on self-reported menopause and age below 60 years at sampling time. Thereby, the women-combined dataset induces large variation of estradiol levels, thus reducing the statistical power in both the GWAS and MR analyses.

The GWAS results of the log-transformed estradiol levels, which we conducted in the UK Biobank cohort study, differed from the analysis of former studies, and by this also the inclusion of genetic instruments in the one-sample MR analyses. The possible reasons for this difference are due to several aspects. Due to the limited information of the menstruation cycle at time of estradiol measurement of the female study participants, we limited the corresponding GWAS to 6,835 self-reported postmenopausal women. The minimum detectable level in the UK Biobank cohort study was 175 pmol/L, thus the detection of estradiol in postmenopausal women was less sensitive compared with other studies like Pott et al. (24). This detection limit affects to a lesser extent the analyses in men, who, on average, have higher estradiol levels than postmenopausal women. In the study of Pott et al., only one locus harboring the signal peptide peptidase-like 2A gene (rs12913657 on chromosome 15) reached genome-wide significance in 4,191 men, but without a replication sample included (24). This locus was not associated with estradiol level in the larger GWAS of the 14,797 men from the UK Biobank dataset. However, our GWAS revealed a highly significant association on chromosome 14 (rs7151019), which was used as instrument in the one-sample MR.

Ruth et al. also conducted a GWAS in the UK Biobank males, but they dichotomized the estradiol level at the detection limit (23). Their GWAS revealed more loci associated with estradiol levels by including a larger sample size. However, the two-sample MR result using this dataset confirmed our one-sample MR. In addition, these eGFR MR results were also directionally consistent with the CKD MR, although not reaching significance after multiple testing. Of note, our significant GWAS locus at chromosome 14 was also revealed by Ruth et al., where one of their top SNPs (rs34019140) was in moderate linkage disequilibrium with our SNP rs7151019 (*R*² = 0.45). However, rs34019140 (or a suitable proxy) was not available in the summary statistics of the kidney traits.

The main strength of our project is the different analyses performed using multiple sources of genetic association data, with two different exposure populations for the use in the large two-sample MR, as well as individual-level data from the UK Biobank cohort study for use in the one-sample analysis. One challenge of the MR approach is avoiding weak instrument bias (31). Thus, we selected only independent variants in the one-sample MR having a strong statistical association with estradiol levels (*p* < 5*10^–8^). Furthermore, it is important that the instruments in a MR are not associated with (unadjusted) confounders of the estradiol–kidney function relationship. Therefore, we conducted the one-sample MR by adjusting for possible confounders, ensuring the robustness of the association results (46, 47).

On the other hand, our study had several limitations. The first is the lack of available estradiol instruments from the meta-analysis results. This shortage is mainly due to the changes in estradiol levels in premenopausal women during the menstruation cycle. Such daily changes make it hard to develop a uniform study design for measuring estradiol levels. An alternative approach is to include exclusively postmenopausal women, which results in a small sample size and is where measurement of estradiol levels is technically difficult. This heterogeneity of estradiol levels in women is the second limitation for our study. The available meta-analysis results for continuous estradiol include populations with relatively small sample sizes, which results in low power for identifying genetic instruments. There was no sample overlap between the estradiol level assessed in the UK Biobank cohort study and eGFR, but the UACR GWAS included the UK Biobank cohort study dataset, which could in turn bias the effect estimates of the respective two-sample MR (48). Finally, no sex-stratified kidney trait GWASs were available, which could be a reason for the non-significant two-sample MR results. Although the MR analyses identified statistically significant associations, the reported effect sizes are small and hard to interpret. However, the effect sizes itself obtained from an MR are generally less informative (31).

Although we found a robust and significant MR result in men, we cannot exclude that there is also a causal effect of estradiol level on kidney function in women. Taking this into account, our results do not allow a conclusion on whether or not the observed differences in kidney disease prevalence between men and women can be attributed to sex-specific differences in estradiol levels.

Our results also highlight the need to identify additional genetic variants associated with estradiol levels in men and women providing instruments for MR analyses, and also in non-European populations. Finding such associations could be challenging, especially in the female population. Furthermore, studies like randomized controlled trials are required to estimate the magnitude of this potential causal relation. Nevertheless, our MR results provide starting points for subsequent studies focusing on the effects of estradiol levels on kidney function which may finally lead to therapeutic strategies as part of preventing kidney diseases.

## Data availability statement

Publicly available datasets were analyzed in this study. The data underlying this article are available from the UK Biobank (GWAS on estradiol), at https://www.ukbiobank.ac.uk/. The remaining datasets were derived from publications as referenced in the manuscript.

## Ethics statement

Ethical approval was not required for the studies involving humans because only published GWAS summary statistics and data obtained from UK Biobank with ethical approval as provided on the study website and in the corresponding publication was used. The studies were conducted in accordance with the local legislation and institutional requirements. The human samples used in this study were acquired from a previous study for which ethical approval was obtained. Written informed consent to participate in this study was not required from the participants or the participants’ legal guardians/next of kin in accordance with the national legislation and the institutional requirements.

## Author contributions

MKN: writing and analysis; AT: supervising, reviewing, writing, and editing; CS: supervising, reviewing, and editing; EB: supervising. All authors contributed to the article and approved the submitted version.
