# Supplementary material for: Mendelian randomization indicates causal effects of estradiol levels on kidney function in males
Source: Front Endocrinol (Lausanne). 2023 Dec 19;14:1232266. doi: 10.3389/fendo.2023.1232266 (PMC10758447; doi:10.3389/fendo.2023.1232266)
Supplement: Supplementary file 1 [file DataSheet_1.pdf]

Mendelian randomization indicates causal effects of estradiol levels on kidney function in males

Mohammed Kamal Nasr *et al.*

SUPPLEMENTAL MATERIAL

Supplementary Tables 3

Supplementary Table 1. Genetic instruments for estradiol in women using the Haas et al. summary statistics 3

Supplementary Table 2. Genetic instruments for estradiol in men using the Ruth et al. summary statistics 4

Supplementary Table 3. Genetic instruments for the Mendelian randomization analysis using eGFR as exposure. 5

Supplementary Table 4. Genetic instruments for the Mendelian randomization analysis using UACR as exposure. 8

Supplementary Table 5. Mendelian randomization analysis results and the heterogeneity in the postmenopausal women for eGFR using Haas et al. summary statistics 10

Supplementary Table 6. Mendelian randomization analysis results and the heterogeneity in the premenopausal women for UACR using Haas et al. summary statistics 10

Supplementary Table 7. Mendelian randomization analysis results and the heterogeneity in the male population using Ruth et al. summary statistics 10

Supplementary Table 8. Study characteristics of the individuals included in the one-sample Mendelian randomization 10

Supplementary Table 9. Results of the Mendelian randomization using estradiol as outcome 11

Supplementary Figures 12

Supplementary Figure 1. Flowchart of the selection of UK Biobank individuals included in the one-sample MR analysis. 12

Supplementary Figure 2: Overview of the analytical steps performed in the one-sample MR analyses. 13

Supplementary Figure 3. Scatter plots of the significant two-sample Mendelian randomization association 14

Supplementary Figure 4. GWAS results for estradiol levels in women 18

Supplementary Figure 5. GWAS results for estradiol levels in the overall sample 19

Supplementary Figure 6. Quantile-Quantile plots of the estradiol GWAS results in the UK Biobank 20

Supplementary Tables

Supplementary Table 1. Genetic instruments for estradiol in women using the Haas et al. *summary statistics*

Overall n=229,966								
Chr	rsID	bp_hg19	E.Allele	A.Allele	Allele.Freq	Estimate	SE	P-value
1	rs2145409	46822998	C	T	0.417	0.018	0.003	1.9E-09
5	rs2454949	176466011	A	G	0.492	0.023	0.003	5.9E-15
6	rs72823349	10950047	T	A	0.266	-0.020	0.003	5.9E-09
8	rs28807105	37893517	G	A	0.227	0.022	0.004	1.0E-09
12	rs75770066	66704225	G	A	0.033	0.062	0.008	5.4E-14
12	rs35997018	130822471	T	C	0.448	-0.018	0.003	2.0E-09
20	rs16991615	5948227	A	G	0.063	0.062	0.006	3.9E-25
Premenopausal n= 51,081								
Chr	rsID	bp_hg19	E.Allele	A.Allele	Allele.Freq	Estimate	SE	P-value
5	rs2454949	176466011	A	G	0.492	0.037	0.006	3.3E-09
12	rs75770066	66704225	G	A	0.033	0.109	0.017	6.9E-11
20	rs16991615	5948227	A	G	0.063	0.102	0.012	1.8E-17
Postmenopausal n= 84,194								
Chr	rsID	bp_hg19	E.Allele	A.Allele	Allele.Freq	Estimate	SE	P-value
5	rs2454949	176466011	A	G	0.492	0.027	0.005	2.4E-08
8	rs28807105	37893517	G	A	0.227	0.034	0.006	9.4E-09
12	rs75770066	66704225	G	A	0.033	0.100	0.014	9.1E-13
20	rs16991615	5948227	A	G	0.063	0.102	0.010	4.1E-24

Chr: chromosome; bp_hg19: variant position (hg19 reference); E.Allele: effect allele; A.Allele: alternative allele; Allele.Freq: effect allele frequency; SE: standard error.

Supplementary Table 2. Genetic instruments for estradiol in men using the Ruth et al. *summary statistics*

n= 206,927								
Chr	rsID	bp_hg19	E.Allele	A.Allele	Allele.Freq	Estimate	SE	P-value
2	rs559555	31810974	T	A	0.440	0.006	0.001	5.7E-11
2	rs112881196	31982811	G	C	0.039	0.025	0.002	5.6E-30
12	rs56196860	2908330	A	C	0.031	0.021	0.002	2.6E-18
15	rs7173595	51533736	T	C	0.649	0.016	0.001	3.6E-72
15	rs3751591	51606710	G	A	0.167	0.008	0.001	6.6E-12
18	rs113047993	20585399	C	T	0.931	0.010	0.002	8.7E-09
19	rs10425629	48384648	C	T	0.165	0.008	0.001	2.7E-11
22	rs117826558*	46770756	T	C	0.034	0.014	0.002	2.7E-08

Chr: chromosome; bp_hg19: variant position (hg19 reference); E.Allele: effect allele; A.Allele: alternative allele; Allele.Freq: effect allele frequency; SE: standard error. *rs117826558 was available only for the CKD Mendelian randomization.

Supplementary Table 3. Genetic instruments for the Mendelian randomization analysis using eGFR as exposure.

n = 567,460

Chr	rsID	bp_hg19	Effect Allele	Alt Allele	Alt Allele Freq	effect	SE	p-value
1	rs11166440	100808363	a	g	0.63	0.002051	0.000359	1.10E-08
1	rs1119066	186658212	a	c	0.15	0.002706	0.000482	1.96E-08
1	rs17413465	55718708	a	c	0.18	0.002518	0.000443	1.36E-08
1	rs1887252	82957871	c	g	0.64	-0.00289	0.000358	7.45E-16
1	rs2490391	243469669	a	c	0.46	-0.0025	0.000346	5.46E-13
1	rs267738	150940625	t	g	0.79	-0.00501	0.000421	1.33E-32
1	rs2792796	56715908	t	c	0.61	-0.00208	0.000352	3.14E-09
1	rs3795503	180905694	t	c	0.33	0.002156	0.000381	1.51E-08
1	rs3850625	201016296	a	g	0.12	0.004806	0.000553	3.57E-18
1	rs509345	150276022	a	g	0.52	0.002443	0.000345	1.35E-12
1	rs6667182	15914545	t	c	0.32	-0.00428	0.00043	2.22E-23
1	rs7543734	94050911	c	g	0.2	0.00312	0.000482	9.58E-11
2	rs10865189	43433257	c	g	0.47	0.002534	0.000356	1.02E-12
2	rs11694902	121988884	a	g	0.14	0.004132	0.000503	2.14E-16
2	rs140179699	120936492	a	g	0.95	0.007306	0.001077	1.16E-11
2	rs1548945	217665788	t	c	0.41	0.003679	0.000359	1.27E-24
2	rs187355703	176993583	c	g	0.97	0.010107	0.001143	9.45E-19
2	rs2301343	40680149	t	g	0.74	-0.0023	0.0004	9.08E-09
2	rs35472707	169995581	t	c	0.05	-0.00754	0.000829	9.53E-20
2	rs3791221	226933	a	g	0.65	0.002138	0.00036	3.00E-09
2	rs6546869	73895765	a	g	0.22	0.006087	0.000416	1.66E-48
2	rs72841902	73372212	a	t	0.29	0.002235	0.000379	3.61E-09
2	rs7565830	159810691	a	g	0.72	-0.00223	0.000387	8.77E-09
2	rs807624	15782471	t	g	0.34	0.003376	0.000363	1.53E-20
3	rs11919484	186432839	t	g	0.31	-0.00232	0.000371	3.98E-10
3	rs66473811	64000464	t	c	0.84	0.003072	0.000483	2.02E-10
3	rs6779368	185298868	a	g	0.66	0.003316	0.000412	8.78E-16
3	rs7651407	48443816	t	c	0.45	0.002651	0.000393	1.56E-11
3	rs9868185	121657593	a	g	0.54	0.002653	0.000345	1.50E-14
4	rs1458038	81164723	t	c	0.3	0.003197	0.00038	3.60E-17
4	rs223308	103812499	a	g	0.52	-0.0027	0.000343	3.00E-15
4	rs28817415	77401452	t	c	0.44	-0.00744	0.000344	9.68E-104
4	rs363092	3196029	a	c	0.42	-0.00218	0.000351	5.55E-10
4	rs71606723	115498457	a	t	0.76	0.002893	0.000405	9.26E-13
5	rs11951093	39421736	a	g	0.42	-0.00558	0.000359	2.05E-54
5	rs12163971	132226669	a	c	0.16	-0.00323	0.000466	4.33E-12
5	rs12520984	52787358	c	g	0.33	0.002192	0.000367	2.37E-09
5	rs13157326	34504277	a	g	0.48	-0.00271	0.000388	2.95E-12

5	rs3797537	78322650	a	g	0.71	0.00212	0.000377	1.86E-08
5	rs3812036	176813404	t	c	0.26	-0.00687	0.000406	3.19E-64
5	rs55938024	67742038	a	g	0.12	-0.00646	0.000605	1.37E-26
5	rs79760705	53298716	t	g	0.11	0.005609	0.000551	2.55E-24
6	rs12207180	160633107	a	t	0.12	-0.00852	0.000537	1.21E-56
6	rs13200335	41690823	a	c	0.42	0.002361	0.00035	1.50E-11
6	rs144100226	34180297	t	c	0.039	0.005993	0.001053	1.26E-08
6	rs3822939	133849789	a	g	0.46	-0.00281	0.000344	3.08E-16
6	rs62432759	154858365	a	g	0.78	-0.00249	0.000432	7.56E-09
6	rs6458868	52630153	t	c	0.65	-0.00213	0.00036	3.50E-09
6	rs6921580	7203714	c	g	0.41	0.002735	0.000356	1.61E-14
7	rs10224002	151415041	a	g	0.72	0.006845	0.000398	2.74E-66
7	rs13230509	1286192	c	g	0.69	-0.00553	0.000434	4.04E-37
7	rs35072105	65609817	a	g	0.55	-0.00211	0.000351	1.97E-09
7	rs4410790	17284577	t	c	0.37	-0.00229	0.000359	1.92E-10
7	rs62491533	129564134	t	c	0.83	-0.00274	0.000458	2.11E-09
7	rs6948759	33095688	t	c	0.21	-0.00258	0.000422	1.01E-09
7	rs700753	46753684	c	g	0.34	0.003295	0.000361	7.50E-20
8	rs2442604	6388533	t	c	0.55	-0.00198	0.000345	9.18E-09
8	rs4871905	23735047	c	g	0.42	-0.0043	0.000346	1.82E-35
9	rs10122824	139109861	t	g	0.34	-0.00239	0.000379	2.84E-10
9	rs2039424	71432174	a	g	0.62	0.004828	0.000361	9.75E-41
10	rs10994860	52645424	t	c	0.19	0.003891	0.000446	2.70E-18
10	rs2068888	94839642	a	g	0.45	-0.00262	0.00035	6.31E-14
10	rs7095954	82209232	a	t	0.47	-0.00191	0.000345	2.82E-08
10	rs80282103	899071	a	t	0.92	0.008084	0.000633	2.58E-37
11	rs11237450	78023356	a	c	0.17	0.003007	0.000502	2.05E-09
11	rs12361687	9890052	a	g	0.36	0.002134	0.000365	5.03E-09
11	rs2156664	121645005	t	c	0.27	-0.00214	0.00039	3.76E-08
11	rs233438	2794392	a	g	0.81	0.004284	0.000441	2.84E-22
11	rs3018667	68912221	a	g	0.32	-0.00238	0.000369	1.23E-10
11	rs3925584	30760335	t	c	0.55	-0.00547	0.000346	3.01E-56
11	rs6484504	31424823	t	c	0.28	-0.00323	0.00039	1.17E-16
11	rs7127946	48250675	t	c	0.72	0.002293	0.000381	1.76E-09
12	rs10846157	15325031	a	c	0.81	-0.00361	0.000437	1.34E-16
12	rs11062167	364739	a	g	0.53	-0.00418	0.000344	7.08E-34
12	rs117113238	12209203	a	g	0.095	0.003939	0.00061	1.06E-10
12	rs7974833	57791833	t	c	0.76	-0.00322	0.00041	3.97E-15
13	rs303937	72372524	a	t	0.41	0.002709	0.000356	2.90E-14
14	rs1028455	88829975	a	t	0.33	0.002062	0.000367	1.90E-08
14	rs2071047	54418411	a	g	0.41	0.001999	0.00035	1.10E-08
14	rs35629566	93072317	c	g	0.83	0.002976	0.00048	5.73E-10
15	rs10851543	53962748	a	g	0.56	0.003048	0.000346	1.34E-18

15	rs10851885	76304503	a	g	0.76	0.004972	0.000408	3.28E-34
15	rs11071738	63580155	t	c	0.53	-0.00249	0.000345	5.49E-13
15	rs11071939	67463391	t	c	0.92	-0.00383	0.000654	4.53E-09
15	rs2472297	75027880	t	c	0.26	0.003857	0.000423	8.21E-20
15	rs4886425	74124543	a	g	0.17	-0.0027	0.00046	4.26E-09
15	rs4886699	75692303	a	c	0.75	0.003142	4.00E-04	4.00E-15
15	rs506000	76817788	t	c	0.91	-0.00383	0.000612	4.17E-10
16	rs113956264	1997004	t	c	0.036	0.008068	0.001198	1.65E-11
16	rs12920176	51761084	a	c	0.59	-0.00262	0.000357	2.35E-13
16	rs28581385	79942679	a	t	0.85	-0.00328	0.000487	1.58E-11
16	rs56140069	69795323	a	t	0.82	0.002529	0.000455	2.67E-08
16	rs7203398	53189672	a	c	0.73	0.002729	0.000391	2.86E-12
16	rs77924615	20392332	a	g	0.2	0.009576	0.000452	1.21E-99
17	rs6501468	66427696	t	c	0.23	0.002399	0.000423	1.37E-08
17	rs9907229	58917399	t	c	0.85	-0.00491	0.000487	6.83E-24
18	rs8096658	77156537	c	g	0.51	0.004558	0.000404	1.77E-29
19	rs111827672	37649866	a	t	0.32	0.003064	0.000369	1.02E-16
19	rs113445505	38157969	t	c	0.37	0.003775	0.000355	1.96E-26
19	rs57126710	37017633	t	c	0.35	0.002468	0.000359	6.18E-12
20	rs1509117	8303120	a	t	0.3	0.002513	4.00E-04	3.33E-10
20	rs1570521	62911019	t	g	0.41	0.002029	0.000352	8.54E-09
20	rs2235826	56143169	a	t	0.81	-0.00328	0.000452	3.94E-13
20	rs6127099	52731402	a	t	0.72	-0.00513	0.000406	1.17E-36
20	rs62187541	1340244	a	g	0.93	-0.00375	0.000684	4.13E-08
21	rs2244237	37818141	t	g	0.22	0.00268	0.000413	8.43E-11
21	rs2823139	16576783	a	g	0.34	-0.00271	0.000365	1.01E-13
21	rs2834317	35356706	a	g	0.15	-0.00309	0.00049	2.83E-10

Chr: chromosome; bp_hg19: variant position (hg19 reference); SE: standard error.

Supplementary Table 4. Genetic instruments for the Mendelian randomization analysis using UACR as exposure.

n = 547,361

Chr	rsID	bp_hg19	Effect Allele	Alt Allele	Alt Allele Freq	effect	SE	p-value
1	rs17035646	10796547	A	G	0.34	0.012	0.002	1.4E-08
1	rs4641276	33760743	T	C	0.25	-0.013	0.002	3.6E-08
1	rs1337526	47965130	A	G	0.20	-0.024	0.003	1.9E-21
1	rs34257409	155131394	T	G	0.40	0.016	0.002	1.6E-15
1	rs16864515	171435542	A	C	0.10	-0.019	0.003	2.0E-08
1	rs78444298	184672098	A	G	0.02	-0.047	0.008	2.8E-10
1	rs3850625	201016296	A	G	0.12	0.018	0.003	1.4E-08
2	rs4665972	27598097	T	C	0.40	0.017	0.002	6.2E-17
2	rs12714144	85754578	A	T	0.87	0.022	0.003	5.5E-14
2	rs2880119	111809330	A	C	0.86	-0.016	0.003	9.2E-09
2	rs10207567	203714973	C	G	0.82	0.019	0.003	3.2E-14
2	rs1047891	211540507	A	C	0.31	-0.019	0.002	2.6E-18
2	rs7597336	227942519	A	G	0.87	-0.020	0.003	2.4E-11
3	rs73065147	46894939	T	C	0.93	-0.026	0.004	1.7E-11
3	rs1010553	52540773	T	C	0.52	0.011	0.002	1.7E-08
3	rs112607182	170027407	T	C	0.08	0.030	0.004	2.0E-13
4	rs13132085	56460085	A	G	0.29	-0.013	0.002	6.3E-09
4	rs10023335	77358987	T	C	0.59	0.014	0.002	9.7E-13
4	rs6535594	149132756	A	G	0.50	0.014	0.002	5.7E-13
5	rs76027714	53275370	A	G	0.92	0.023	0.004	1.0E-09
5	rs1309546	64290004	T	C	0.55	0.012	0.002	5.9E-10
5	rs162890	131623658	T	C	0.33	0.013	0.002	6.3E-10
6	rs2240060	31114900	A	G	0.29	0.014	0.002	6.5E-10
6	rs1544935	39124448	T	G	0.78	-0.017	0.002	1.0E-12
6	rs3734692	43817791	A	T	0.69	-0.018	0.002	4.5E-16
7	rs4410790	17284577	T	C	0.37	-0.022	0.002	2.1E-26
7	rs2023844	27243238	A	G	0.93	0.027	0.004	2.3E-12
7	rs17158386	29805361	A	G	0.26	0.020	0.002	2.2E-17
7	rs35692677	69902654	A	G	0.19	-0.016	0.003	2.9E-10
7	rs1057868	75615006	T	C	0.28	0.012	0.002	3.1E-08
8	rs7812843	23737080	A	G	0.50	-0.012	0.002	3.6E-09
8	rs4738817	61620613	A	G	0.45	-0.012	0.002	7.4E-09
8	rs6998967	81364205	A	G	0.17	-0.015	0.003	1.4E-08
8	rs2954021	126482077	A	G	0.49	0.015	0.002	6.9E-14
10	rs74375025	16947664	A	G	0.11	0.038	0.003	4.2E-28
10	rs2793351	22151578	A	G	0.69	0.012	0.002	2.2E-08
10	rs67339103	77893686	A	G	0.22	0.017	0.002	2.5E-12
10	rs2068888	94839642	A	G	0.45	-0.012	0.002	5.5E-10

11	rs113139575	10296221	C	G	0.94	-0.025	0.004	1.2E-09
11	rs988712	27563382	T	G	0.24	-0.013	0.002	1.8E-08
11	rs7115200	71752160	T	G	0.56	-0.012	0.002	2.0E-09
11	rs12790943	120058623	T	C	0.42	0.014	0.002	1.2E-11
12	rs2601006	69979517	T	C	0.34	-0.015	0.002	1.6E-13
14	rs11158763	69253343	T	C	0.46	-0.013	0.002	1.3E-11
15	rs3784283	41867782	A	T	0.60	0.015	0.002	8.9E-14
15	rs2433611	45665653	A	C	0.26	-0.018	0.002	9.1E-15
15	rs146311723	63804507	T	C	0.82	-0.015	0.003	6.6E-09
15	rs2470893	75019449	T	C	0.33	0.023	0.002	1.1E-26
17	rs11078597	1618363	T	C	0.81	-0.016	0.003	7.1E-10
17	rs677888	37461018	T	G	0.76	-0.014	0.002	7.3E-10
17	rs35572189	79419025	A	G	0.36	-0.012	0.002	3.0E-08
18	rs11659764	53335512	A	T	0.05	0.030	0.004	1.8E-11
19	rs1688031	35556640	T	C	0.14	-0.019	0.003	1.6E-11
19	rs143200968	41338847	C	G	0.02	-0.039	0.007	2.1E-08
19	rs15052	41813375	T	C	0.83	0.017	0.003	1.6E-10
19	rs838142	49252151	A	G	0.72	0.017	0.002	8.8E-14
20	rs6119771	30770375	C	G	0.43	0.011	0.002	1.9E-08
22	rs11912350	30748027	T	C	0.76	-0.013	0.002	2.4E-08

Chr: chromosome; bp_hg19: variant position (hg19 reference); SE: standard error.

Supplementary Table 5. Mendelian randomization analysis results and the heterogeneity in the postmenopausal women for eGFR using Haas et al. *summary statistics*

MR Analysis Result					Heterogeneity	
Method	#SNPs	Estimate	Standard error	P-value	Egger intercept	P-value
MR Egger	4	0.011	0.010	0.341	0.919	0.632
Weighted median	4	0.010	0.006	0.090	-	-

Supplementary Table 6. Mendelian randomization analysis results and the heterogeneity in the premenopausal women for UACR using Haas et al. *summary statistics*

MR Analysis Result					Heterogeneity	
Method	#SNPs	Estimate	Standard error	P-value	Egger intercept	P-value
MR Egger	3	-0.055	0.057	0.515	0.082	0.774
Weighted median	3	-0.041	0.031	0.187	-	-

Supplementary Table 7. Mendelian randomization analysis results and the heterogeneity in the male population using Ruth et al. *summary statistics*

MR Analysis Result					Heterogeneity	
Method	#SNPs	Estimate	Standard error	P-value	Egger intercept	P-value
MR Egger	7	0.148	0.038	0.011	3.925	0.560
Weighted median	7	0.076	0.020	1.9E-04	-	-

Supplementary Table 8. Study characteristics of the individuals included in the one-sample Mendelian randomization

**Parameter**	**Count / Median**
Number of included individuals	11,798
Age (Years)	58 [50-64]
BMI (kg/m^2)	27.7 [25.3-30.7]
Estradiol (pmol/L)	204 [188.8-231.2]
UACR (mg/g)	7.665 [5.142-12.77]
GFR (ml/min/1.73 m^2)	95.047 [87.274-102.178]
CKD risk	803 (6.81 %)

For dichotomous data percentage, and for continuous data [1st-3rd quartile] are presented.

Supplementary Table 9. Results of the Mendelian randomization using estradiol as outcome

Group	Exposure	#SNPs	Estimate	95% CI	P-value	Q pval
female	eGFR	108	-0.437	-1.227, 0.353	0.278	0.028
	UACR	58	0.060	-0.308, 1.36	0.491	0.129
male	eGFR	108	0.027	-0.147, 0.319	0.704	0.863
	UACR	58	-0.020	-0.308, 1.36	0.428	0.196

Q pval represents the heterogeneity test result.

Supplementary Figures

Supplementary Figure 1. Flowchart of the selection of UK Biobank individuals included in the one-sample MR analysis.

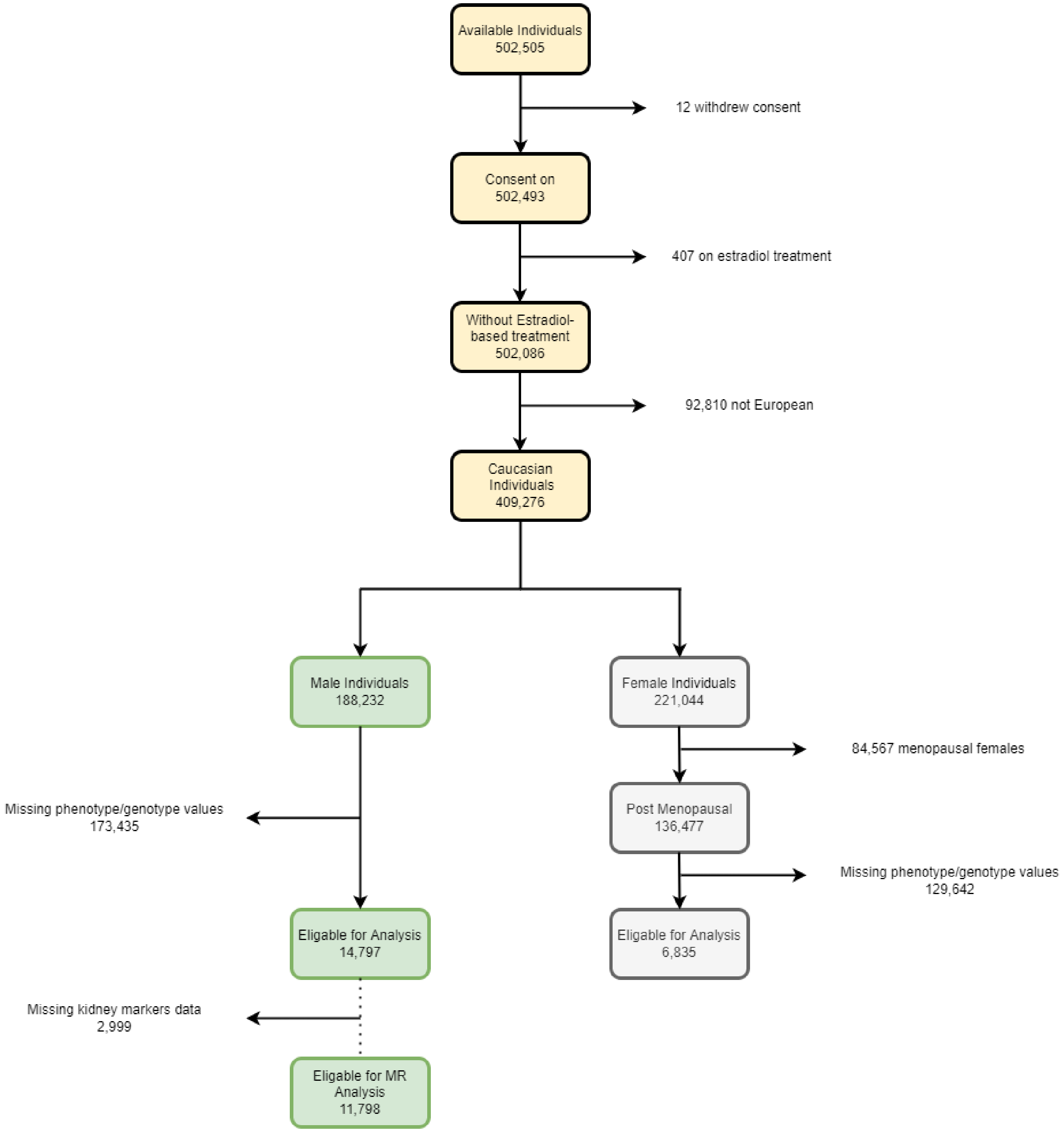

Supplementary Figure 2: Overview of the analytical steps performed in the one-sample MR analyses.

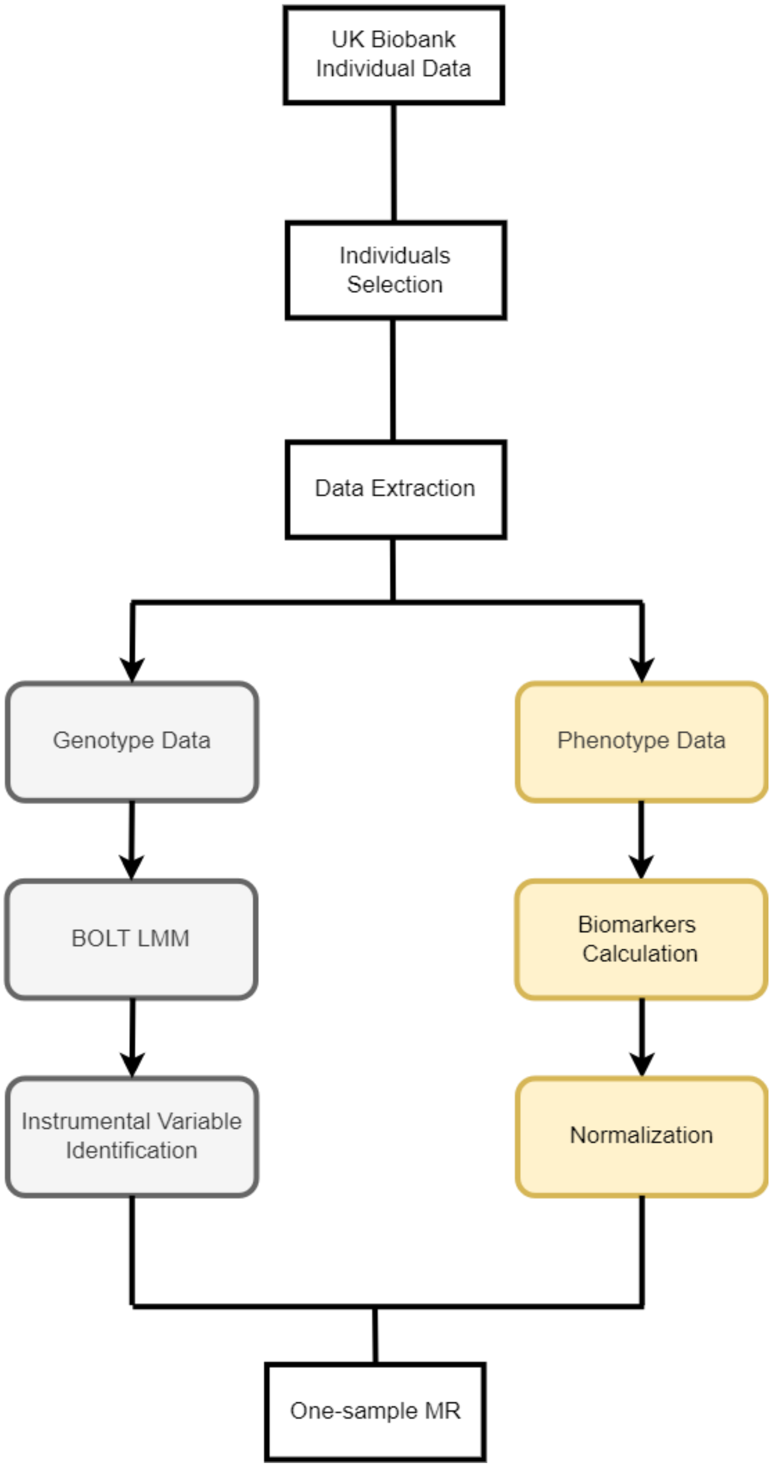

Supplementary Figure 3. Scatter plots of the significant two-sample Mendelian randomization association

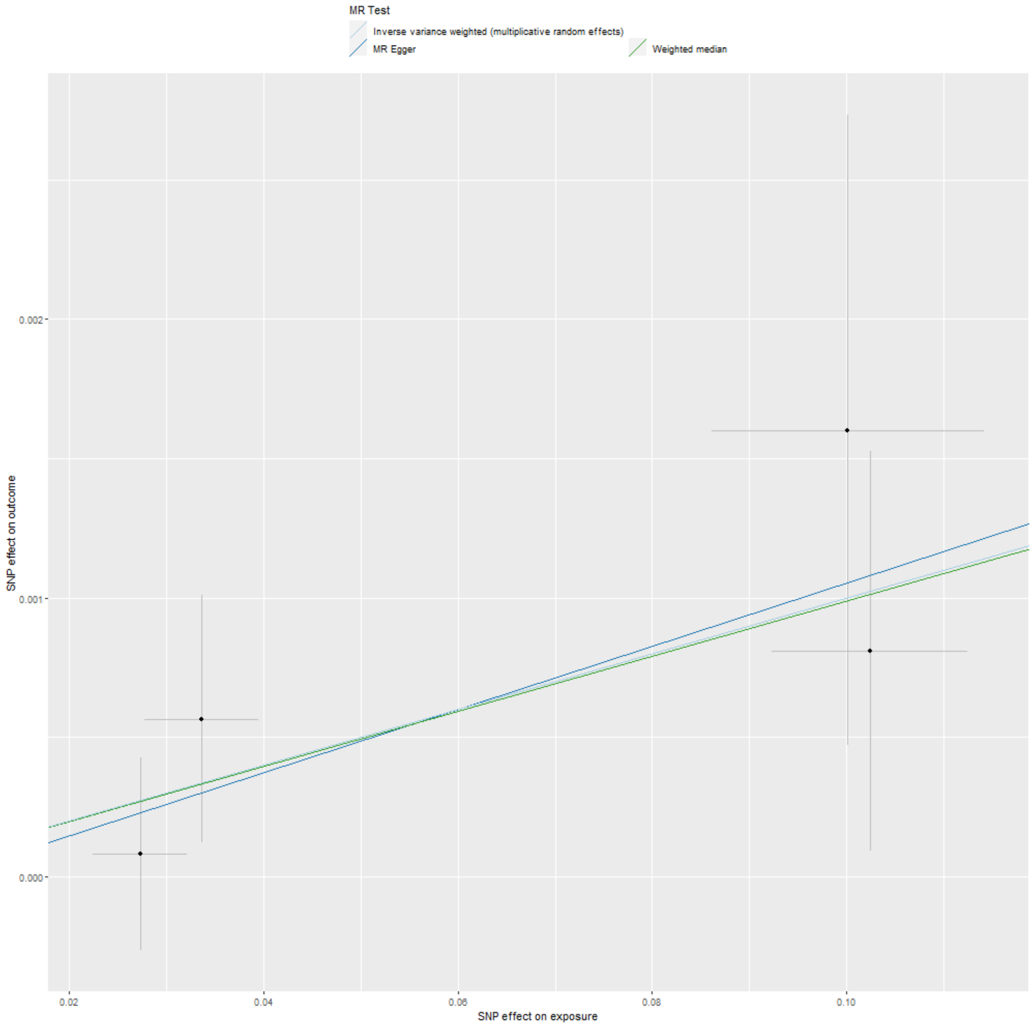

A) Scatter plot of the effect of each instrumental SNP on estradiol vs. eGFR using Haas *et al.* (postmenopausal women) dataset. Each line's slope represents the estimated Mendelian randomization effect per method.

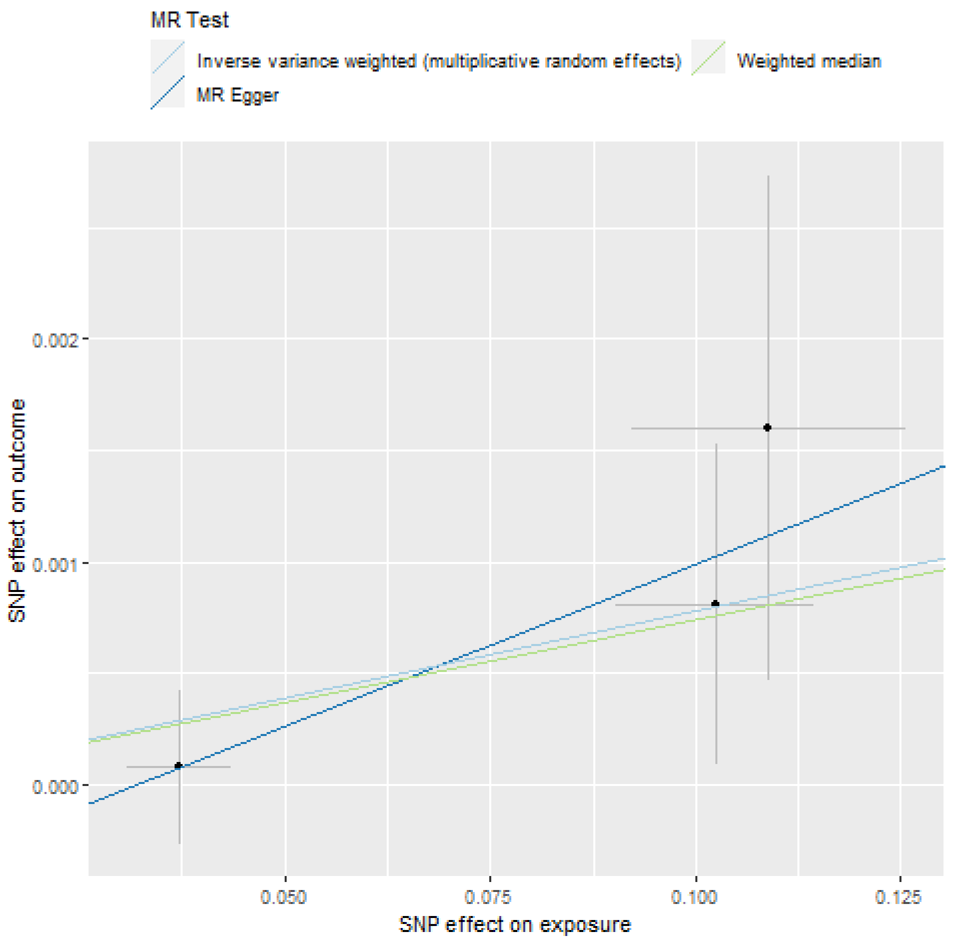

B) Scatter plot of the effect of each instrumental SNP on estradiol vs. eGFR using Haas *et al.* (premenopausal women) dataset. Each line's slope represents the estimated Mendelian randomization effect per method.

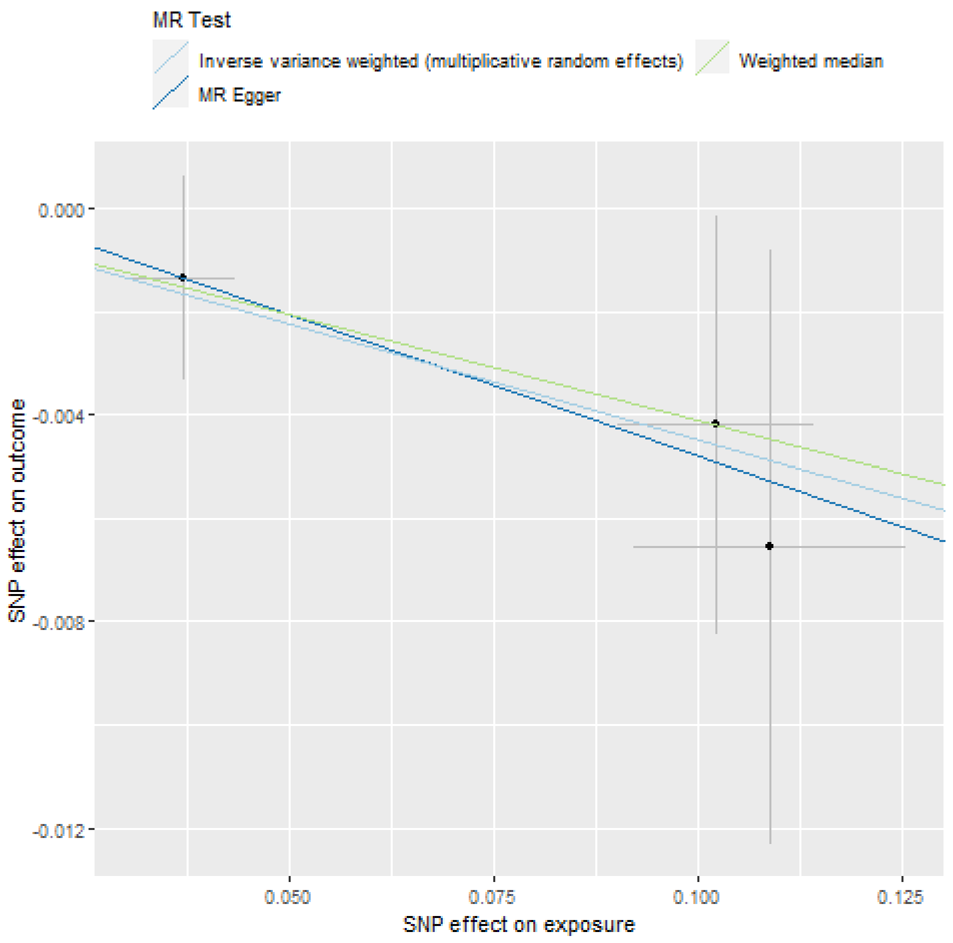

C)

Scatter plot of the effect of each instrumental SNP on estradiol vs. UACR using Haas *et al.* (postmenopausal women) dataset. Each line's slope represents the estimated Mendelian randomization effect per method.

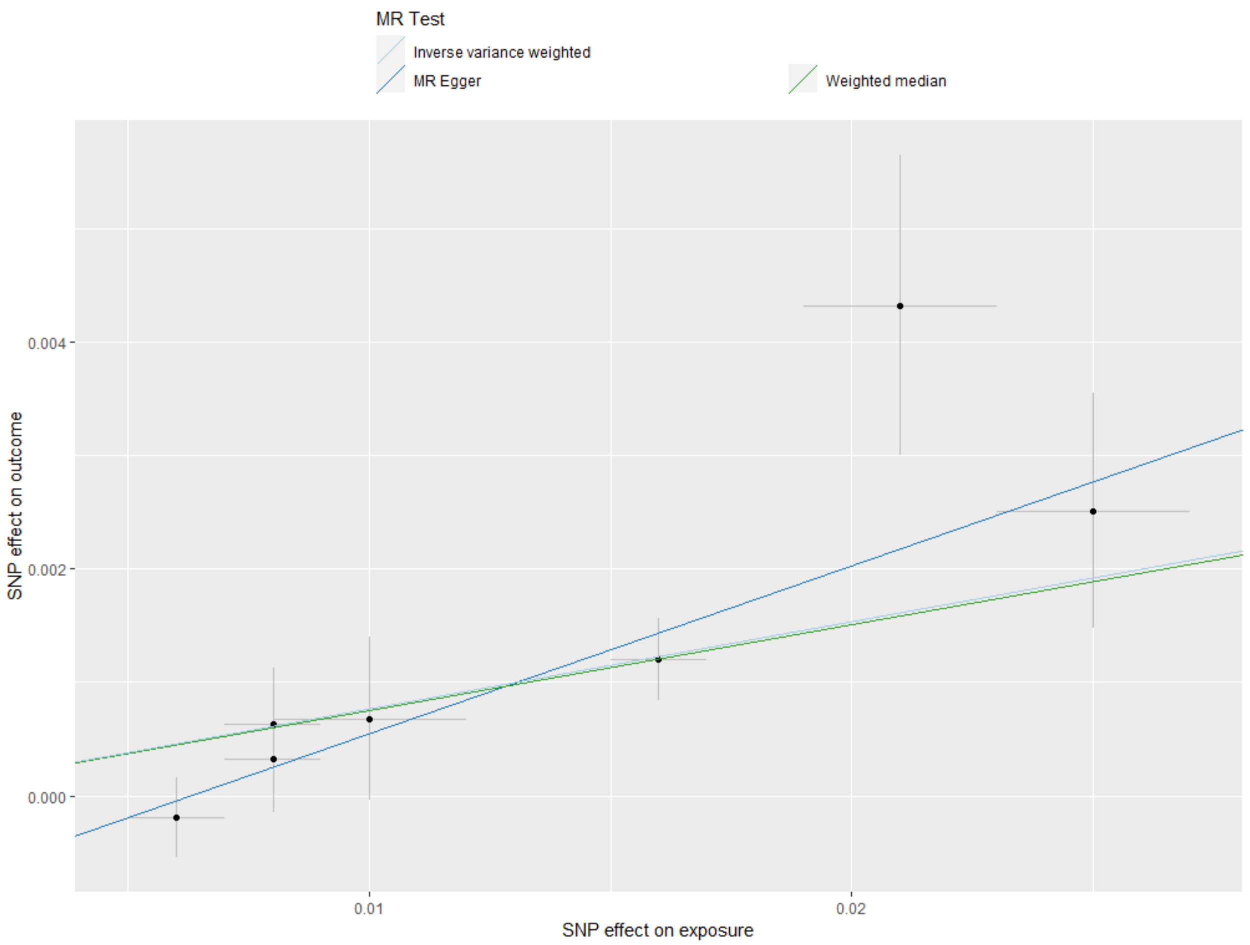

D) Scatter plot of the effect of each instrumental SNP on estradiol vs. eGFR using Ruth *et al.* (male) dataset. Each line's slope represents the estimated Mendelian randomization effect per method.

Supplementary Figure 4. GWAS results for estradiol levels in women

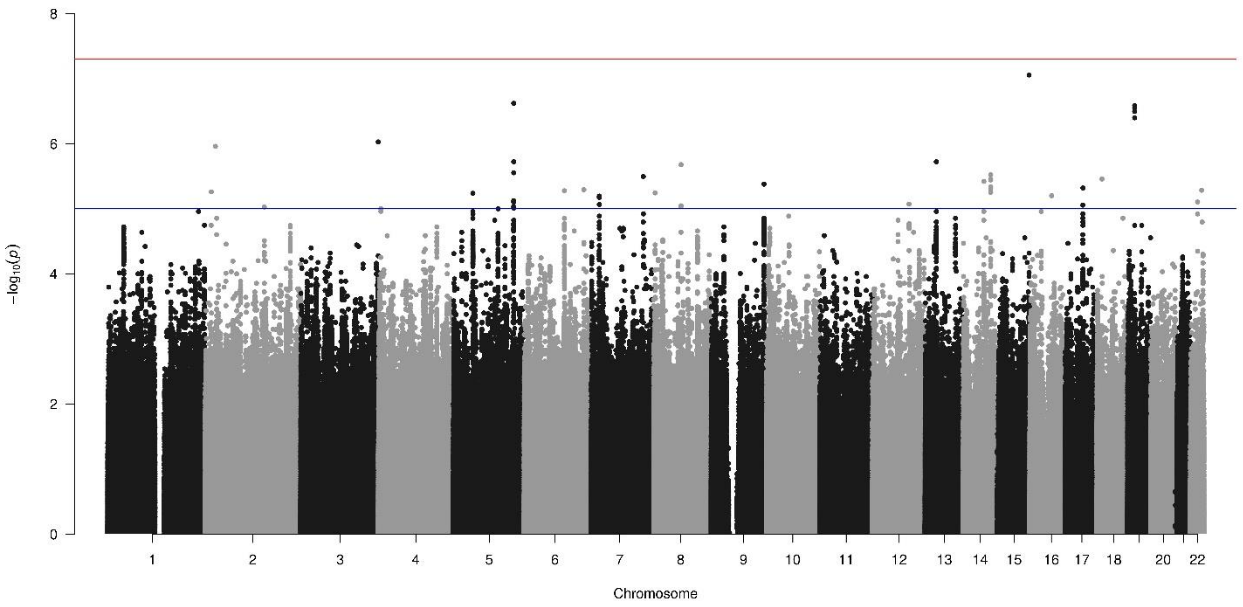

Manhattan plot showing the SNP positions on the x-axis, and their association $-\log_{10}$ (p-values) on the y-axis. The red line represents the threshold for genome-wide significance ($5*10^{-8}$), and the blue line a suggestive significance threshold of 10^{-6}.

Supplementary Figure 5. GWAS results for estradiol levels in the overall sample

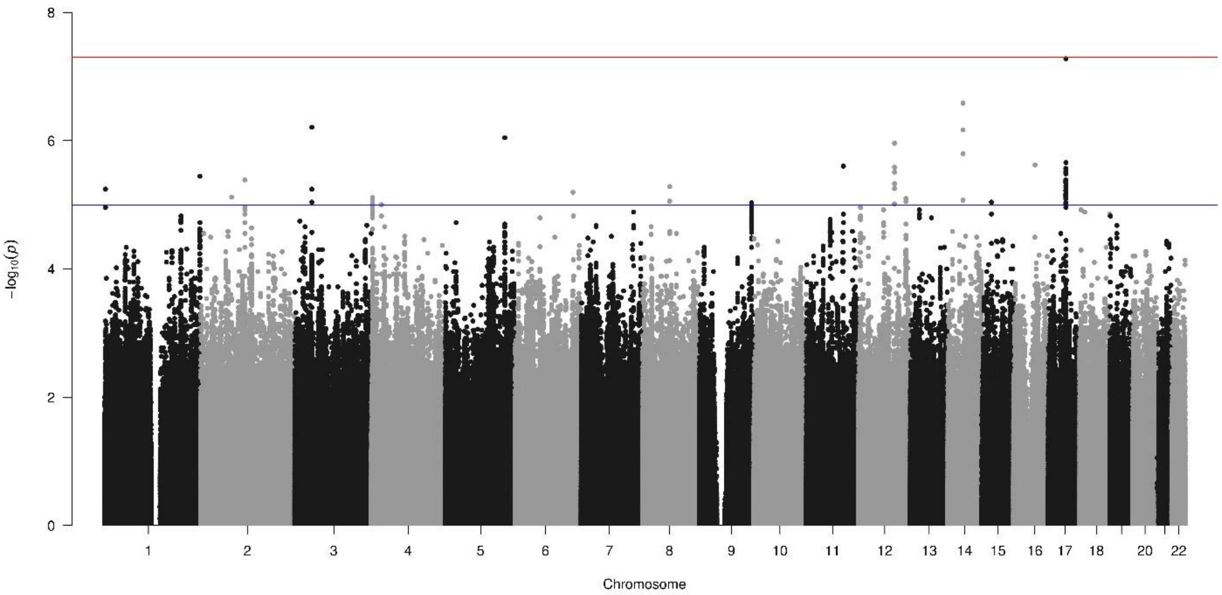

Manhattan plot showing the SNP positions on the x-axis, and their association $-\log_{10}$ (p-values) on the y-axis. The red line represents the threshold for genome-wide significance ($5*10^{-8}$), and the blue line a suggestive significance threshold of 10^{-6}.

Supplementary Figure 6. Quantile-Quantile plots of the estradiol GWAS results in the UK Biobank

A

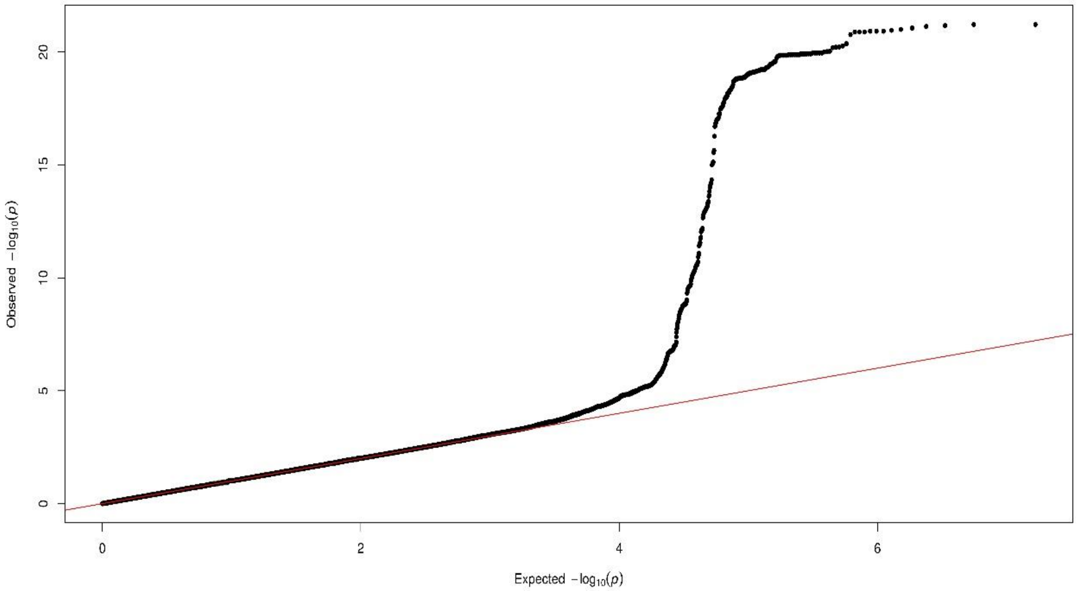

B

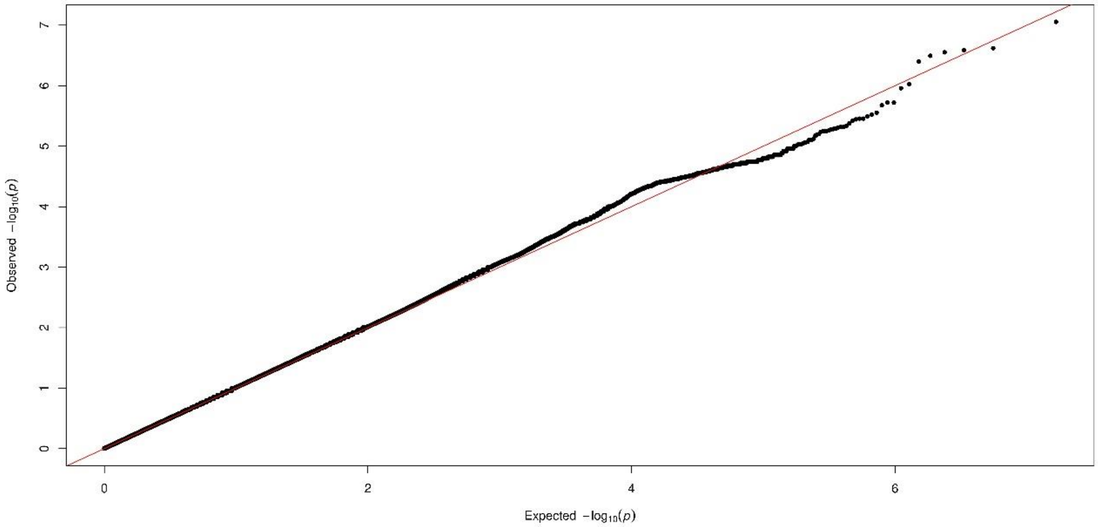

C

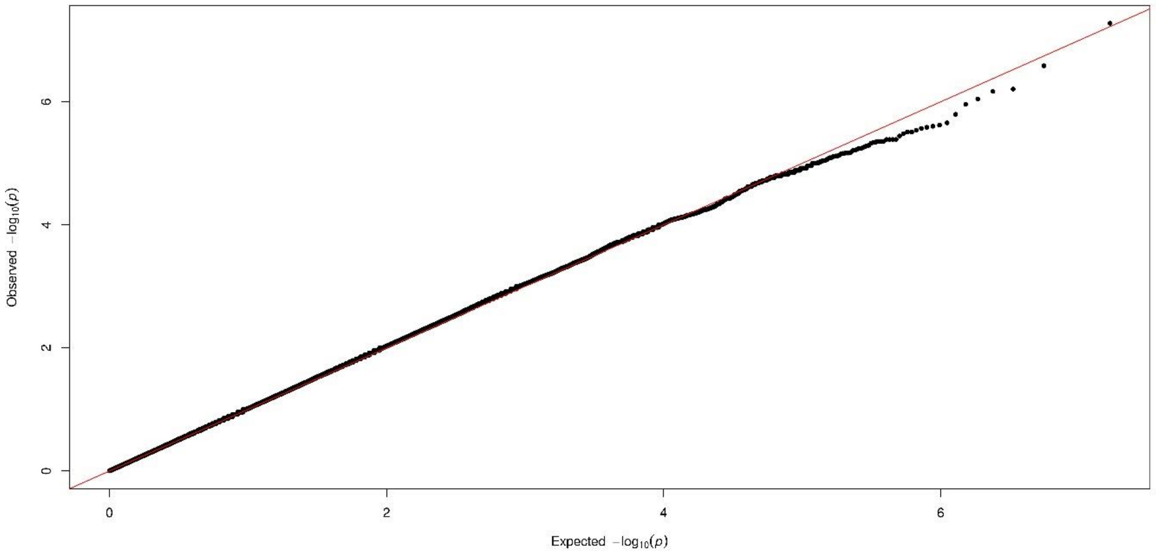

Quantile-Quantile plot of the (**A**) men, (**B**) women, and (**C**) overall GWAS presenting the p-values on a -$\log_{10}$ scale.
